# Physical education, school physical activity, school sports and academic performance

**DOI:** 10.1186/1479-5868-5-10

**Published:** 2008-02-25

**Authors:** François Trudeau, Roy J Shephard

**Affiliations:** 1 Department of Physical Activity Sciences, Université du Québec à Trois-Rivières, Trois-Rivières, Quebec, Canada; 2 Faculty of Physical and Health Education, and Dept. of Public Health Sciences, Faculty of Medicine, University of Toronto, Toronto, Ontario, Canada

## Abstract

**Background:**

The purpose of this paper is to review relationships of academic performance and some of its determinants to participation in school-based physical activities, including physical education (PE), free school physical activity (PA) and school sports.

**Methods:**

Linkages between academic achievement and involvement in PE, school PA and sport programmes have been examined, based on a systematic review of currently available literature, including a comprehensive search of MEDLINE (1966 to 2007), PSYCHINFO (1974 to 2007), SCHOLAR.GOOGLE.COM, and ERIC databases.

**Results:**

Quasi-experimental data indicate that allocating up to an additional hour per day of curricular time to PA programmes does not affect the academic performance of primary school students negatively, even though the time allocated to other subjects usually shows a corresponding reduction. An additional curricular emphasis on PE may result in small absolute gains in grade point average (GPA), and such findings strongly suggest a relative increase in performance per unit of academic teaching time. Further, the overwhelmingly majority of such programmes have demonstrated an improvement in some measures of physical fitness (PF). Cross-sectional observations show a positive association between academic performance and PA, but PF does not seem to show such an association. PA has positive influences on concentration, memory and classroom behaviour. Data from quasi-experimental studies find support in mechanistic experiments on cognitive function, pointing to a positive relationship between PA and intellectual performance.

**Conclusion:**

Given competent providers, PA can be added to the school curriculum by taking time from other subjects without risk of hindering student academic achievement. On the other hand, adding time to "academic" or "curricular" subjects by taking time from physical education programmes does not enhance grades in these subjects and may be detrimental to health.

## Background

The purpose of this paper is to review relationships between physical education (PE), school physical activity (PA), school sports and academic performance. These relationships have been the subject of extensive discussion between advocates and skeptics of PE, school PA and school sports programmes. Both elements of this discussion (academic achievement and physical activity) are independent determinants of a child's health. Our intent in this article is to assess the effects on academic achievement of school PA programmes (including PE and school sports), in both elementary and high schools. Previous reviews have examined relationships between PA and academic achievement. [1-4] Recent research results, echoed in the media, suggest that such activity may have a positive impact on learning and memory. It is now fairly well-recognized that PA is associated with the maintenance of cognitive function in older adults [5] and offers some protection against Alzheimer's disease. [6] Cognitive dysfunctions in older adults is becoming an urgent public health problem, given the ever-rising average life expectancy and the associated growth in the proportion of old and very old individuals in most societies. A positive association between PA and cognitive health is also suspected in younger subjects, but is not as well documented in this age group. Nevertheless, any positive influence of PA on the cognitive functions of children is important for at least 2 reasons: 1) It is a potential argument for increasing PE and/or other types of school PA without risk of decreasing academic progress, and 2) It may offer a way to reduce disruptive behaviour at school and the drop-out from educational programmes. Furthermore, an important by-product of an increased participation to school PA would be an enhanced level of physical fitness.

## Methods

### Search methods

The databases searched included MEDLINE (1966 to 2007), PSYCHINFO (1974 to 2007), SCHOLAR.GOOGLE.COM, and ERIC, as well as the extensive personal databases of the authors. The reference lists of the articles thus identified were also consulted to identify additional potentially-relevant research. Publications in languages other than English were considered where appropriate. For the purpose of this review, we use the term academic achievement to encompass academic success, school performance and all combinations of these terms.

The outcomes of school PA/PE and academic achievement, success or performance were actual or self-reported grade point average (GPA) and determinants of GPA that could potentially be changed by the interventions (concentration, learning, classroom behaviour, engagement in learning, self-esteem, etc.). The terms physical education, physical activity and sports are, for the purposes of this review, restricted to programmes offered within the school context (i.e. instructional physical education and extracurricular physical activity, including in-school physical activity programmes, intraschool and intramural sport).

## Results

### Quasi-experimental and longitudinal studies

It is not surprising that no randomized controlled trials were identified, as they are not practicable in this type of research setting. Quasi-experimental protocols are usually indicated when causality cannot be tested by a random controlled trial in milieux such as the school setting. Seven quasi-experimental studies were identified (Table 1). Cross-sectional studies were also considered, as well as experimental or laboratory experiments on the determinants of academic performance (i.e. learning concentration, classroom behaviour, etc.).

**Table 1:** Quasi-experimental studies examining the influence of sport, physical education or physical activity upon academic achievement.

Author	Sample	Milieu	Intervention	Outcome measure	Response
Fourestier [7]	Children in final year of primary school (13 years), 1 class, n = ?	Vanves (Paris)	Various sports and other activities, 13 h/wk increase for one year	Overall academic performance	Enhanced in experimental group
Shephard et al. [8]	546 children in grades 1 through 6	Trois Rivières, Québec	5 h of specialist physical education per week for 6 years	Teacher ratings, Standard Provincial examination, WISC tests	Enhanced teacher ratings, Maths but not English improved in Provincial exams, 3–4% gain on WISC
Sallis et al. [9]	655 children grades 5 and 6	California	27–42 min additional physical education per week for two years	Metropolitan achievement tests	Non-significant trend to gains in English, arithmetic and behaviour
Dwyer et al. [11]	500 10-year-old students	South Australia	75 min/day of endurance training	Scores for reading and arithmetic	Non-significant trend to gains in English and arithmetic at 2-year follow up
Ahamed et al. [13]	287 9–11 year old primary students	British Columbia	Added 47 min/wk of varied activities for 16 months	Canadian Achievement Test (CAT-3)	Slight trend to improved scores
Coe et al. [14]	214 grade 6 students	Western Michigan, U.S.A.	Nominal 55 min/day (actual 19 min/day) physical education for one semester	Classroom assessments and nationally standardized achievement scores	No change in academic performance except in sub-group who exercised vigorously
Raviv et al. [15]	358 kindergarten and grade 1 students	Israel	One-year movement education program	Reading skills and arithmetic skills	Both improved relative to controls

The first documented quasi-experimental study relevant for to this paper was the Vanves (France) investigation; this involved a small group of schoolchildren tested during the 1950's. [7] Schoolchildren who spent mornings in the classroom and afternoons doing PE were said to perform better academically than children from a control class, but no further details were given. [7] Unfortunately, the specifics of these observations were not described in peer reviewed journals.

A second quasi-experimental study conducted in the Trois-Rivières region (Québec) between 1970 and 1977 involved 546 primary school students; this noted that students involved in an experimental 5 hours of physical education per week had a higher academic performance than their control counterparts who were enrolled in the normal school program for 40 min per week [8]. The supplemental 260 minutes allocated to PE was necessarily taken from time for other academic teaching (i.e. an average 14% curtailment of academic instruction). Despite this curricular change, during the last 5 years of primary school, the overall academic performance of the experimental students improved relative to the controls. During standardized Provincial examinations, children receiving the 5 hours/week of PE had higher scores in mathematics, but lower scores in English (their second language), despite the fact that 33 minutes were removed from mathematics instruction and none from English. [3]

A 2-year quasi-experimental study followed 759 Californian children in the 5th and 6th grades. [9] Subgroups of children were taught PE by either a professional physical educator (n = 178), a trained homeroom teacher (n = 312), or in the normal programme (n = 165). The professional physical educators, the trained teachers, and normal programmes offered, respectively, 80, 65, and 38 minutes per week of PE. As expected, those taught by the professional physical educators achieved greater fitness (cardiovascular and muscle endurance). [10] Also, the groups taught by the professional physical educators and trained teachers had smaller declines in academic performance despite allocating more time to PE. Four of 8 statistical comparisons disclosed an advantage for students in the experimental groups; one comparison was advantageous to control students, while the remaining 3 were equal. The group who spent the most time on PE (i.e. those with a professional physical educator) showed no negative effects on academic achievement and indeed the decline of academic results during the 2 years of the intervention was smaller than that observed in the control subjects. [9]

In South Australia, the 500-student SHAPE trial added 1.25 hours per day of endurance fitness training to the curriculum of 10-year-old primary school students. [11] Over the first 14 weeks of the study, the experimenatl group showed gains in physical work capacity and decreases in body fat relative to controls. Arithmetic and reading scores were not adversely affected by the substantial reallocation of curricular time in favour of PE. These physical benefits appeared to be maintained over the succeeding 2 years in a follow-up of 216 participants. These follow-up evaluations showed (non-significant) trends for better arithmetic and reading grades in experimental students, as well as beneficial changes in teachers' ratings of classroom behaviour. [12]

The 16-month Action School BC! project involved a population of 287 British Columbian primary school children (4th and 5th years: 9–11-years olds). PA was delivered by classroom teachers, amounting to 47 minutes more per week in interventional than in control schools (139 ± 62 vs. 92 ± 45 minutes, *P *< 0.001). [13] Despite a corresponding decrease in academic time, the academic performance of the experimental group, as measured by the Canadian Achievement Test, remained unchanged; indeed, data analysis revealed a trend towards an enhanced academic performance in the intervention schools (the average score rising from 1,595 to 1,672 units).

Another interventional study of 6^th ^grade (11 year-old) students covered a single school term. Fifty-five minutes/day of PE were included in the curriculum, vs. the same allocation of time for arts or computer sciences; the two groups performed equally well in mathematics, sciences and English. [14] Finally, an intervention in Israel involved 92 preschool and 266 first grade children. [15] The experimental manipulation here was a school-based movement education programme, and children in the experimental group showed greater reading skills and arithmetic scores than controls. [15]

Taken together, these quasi-experimental data suggest that the enriched PE programmes demanded a substantial reduction in the time allocated for academic tuition. Since the children achieved at least equally despite the reduced teaching time, the evidence seems strong that the efficiency of learning was enhanced. [3] Despite the variety of programme durations and locations, a common and valuable by-product was a significant increase in various measures of physical fitness (PF).

### Cross-sectional studies

Cross-sectional studies commonly have difficulty in controlling for potential biases, particularly socio-economic status (SES). SES remains the strongest predictor of academic achievement [16] and is also one of the strongest predictors of PA participation in children (e.g. in Canada [17]; Italy [18] and Estonia [19]). Cross-sectional studies generally indicate a positive association with academic achievement. Some of these studies did control for confounders such as SES, and still most of them found a positive association between physical activity and academic achievement (Table 2).

**Table 2:** Cross-sectional studies examining the influence of sport, physical education or physical activity upon academic achievement.

Author	Sample	Milieu	Outcome measure	Response
**Significant association**				

Nelson and Gordon-Larsen [20]	US National Longitudinal Study of Adolescent Health	USA	Grades	Risk ratio for higher results 1.20 for mathematics and 1.21 for English
Field et al. [21]	52 girls and 37 boys in suburban high school	USA	GPA	Higher GPA
Dwyer et al. [24]	7961 schoolchildren, aged 7–15 years in 109 schools	Australia	Rating by the school principal	school performance positively associated with PA in the preceding week
Pate et al. [25]	Youth Risk Behavior Survey adolescents	USA	Perception of academic performance	Inverse relation with level of PA.
Williams [26]		England	GPA	positive association between school sports participation and AA
Sigfudsdottir et al. [27]		Iceland	self-reported school performance	r = -0.11 with absenteeism and r = 0.09 with grades
**Negative or null outcomes on AA**				
Tremblay et al. [28]	6,923 grade 6 children	New Brunswick (Canada)	GPA and self-esteem	Inverse relation PA and AA
Daley and Ryan [29]	232 boys and girls (13–16 years old)	England	self-reported PA and GPA	No relationship except for the duration of PA time vs. marks for English (r = -0.29 to -0.30)
Dollman et al. [30]	Primary school children grades 3, 5 and 7 in 117 schools	Australia	Reading and maths scores	No relation
Yu et al. [31]	333 Chinese pre-adolescents (aged 8–12)	Hong Kong, China	Examination results and conduct grades	No relation with AA but relation with self-esteem

#### Positive results on GPA

Nelson and Gordon-Larsen [20] analyzed results from the US National Longitudinal Study of Adolescent Health; they observed that adolescents who were active in school were more likely to have high grades. Even after adjustment for demographics and SES, the risk ratio of higher grades was 1.20 for mathematics and 1.21 for English among adolescents who were active at school. Within middle to upper middle SES categories, a cross-sectional study of suburban high school seniors (52 girls and 37 boys) found that the more active group had higher GPA. [21]

4,690 Hong Kong children from primary 5 to secondary 7 (i.e. grades 5 to 12) completed a pre-validated questionnaire relating their sports and exercise participation to perceived academic performance. [22] Low correlations were seen for the whole sample (r = 0.10, P < 0.01; r = 0.17, P < 0.01 for females; r = 0.06, NS for males). GPA was not a significant correlated with PA participation when all school bands were confounded; however, the high band showed a positive link between GPA and PA participation, whereas students in the low band showed a negative relationship between PA participation and GPA. [23] These reports suggest that the relationship between PA and academic performance is influenced by the type of students and/or the school that they attend. Deliberate stratification of students by learning ability is by no means universal, but we cannot exclude the possibility that spontaneous, unplanned banding may also influence the strength of observed relationships.

Dwyer et al. [24] made a cross-sectional survey of 9000 Australian schoolchildren between the ages of 7 and 15 years (500 in each age/sex stratum drawn from 109 schools, i.e. 10 girls and 10 boys per school). Depending on the group, a linear regression analysis with good control of confounding variables demonstrated a significant association between academic achievement and PA (a combination of lunchtime PA and minutes of PA the preceding week). In all subjects aged 9–12 years, school performance was positively associated with ratings of PA during the preceding week. In girls 10–15 years old and boys 8–15 years old, academic achievement was also positively associated with the estimates of lunchtime PA. The correlation coefficients between PA and academic achievement, although low (r = 0.08 to 0.19) were statistically significant, suggesting that PA was contributing to academic achievement in both boys and girls. Data from the Youth Risk Behavior Survey likewise showed that a perception of little or no involvement in PA was associated with a perception of low academic performance. [25] Another cross-sectional study from England also controlled for SES; this again reported a positive association between school sports participation and academic achievement. [26]

Researchers from Iceland designed a study included other health behaviours. [27] They found small but significantly positive univariate associations of PA with self-reported school performance (r = -0.11 with absenteeism and r = 0.09 with grades). When confounders were considered, these associations were further weakened, but nevertheless remained statistically significant predictors if selected health behaviours and psychological variables were included in the prediction model. [27]

#### Negative or null outcomes on GPA

In 6,923 grade 6 New Brunswick children (age 11 years), PA showed a weak inverse association with academic achievement, but a positive association with self-esteem. [28] A study on 232 English boys and girls (13–16 years old) found no relationship between self-reported PA and GPA. Moreover, in children aged 13, 14, or 16 years, the durartion of PA was negatively correlated with marks for English (r = -0.29 to -0.30). [29] To our knowledge, these are the only 2 studies to observe negative associations between PA (but not PE) and academic achievement.

A survey of 117 Australian primary schools found no deterioration of literacy and numeracy results in primary school grades 3, 5 and 7 when more time was allocated to PE. [30] SES was the strongest predictor of both literacy and numeracy scores. A recent analysis of Hong Kong pre-adolescent boys reported that a high level of PA at school was associated with high self-esteem, but not with academic achievement. [31]

Even studies that failed to find a positive relationship between PA/PE and GPA have generally found no decrease in academic achievement as a consequence of increased participation in PA (Table 2). Clearly, the absence of an elevation in GPA should not be interpreted as a negative outcome. This is well illustrated by a survey conducted in Virginia's primary schools. [32] A reduction in the time allocated for PE (or the arts) did not improve performance in other subjects like mathematics or reading. Moreover, increasing the time allocated to PE (or the arts) at the expense of other academic subjects was not detrimental to test scores in these subjects. [32] Taken together, these observations suggest that if academic achievements are maintained while spending less time on a specific discipline, the intervention has increased academic efficacy.

### Effects of PA on elements considered to favour academic performance

Many factors like classroom behaviour, self-esteem, self-image, school satisfaction and school connectedness have been postulated as determinants of academic achievement.

#### Classroom behaviour

Self-identification as a school athlete vs. a «jock» is associated with a lower rate of reported misconduct at school [33], with the exception of binge drinking. [34] In the American linguistic context, the word "jock" refers to an individual whose life is oriented toward sport; it is not necessarily a pejorative term. However, it should not be confused with the focused and planned life of a typical athlete.

In the Trois-Rivières study, competencies linked to behaviour were similar overall in the experimental vs. the control group. [35] A German cross-sectional study (CHILT) compared 12 intervention schools (n = 668) vs. 5 control schools (n = 218), finding that PF was associated with concentration in 6–7 years old children. [36]

Evans et al. [37] reported a lower rate of inappropriate talking among emotionally, or behaviourally-disturbed children who were participating in a jogging and football exercise programme. Furthermore, a meta-analysis on the effect of exercise prior to classes led to the conclusion that most exercise interventions significantly reduced disruptive behaviours in disturbed students. [38] These effects could reflect in part better teacher attitudes towards these children, as seen in the Trois-Rivières [3] and the Australian [1] quasi-experimental studies.

#### Other psychosocial effects

Better self-esteem or self-image [20,39] and body image [40] are commonly associated with high levels of PA. Many studies have also linked school sport or PA programmes with other psychosocial outcomes, such as school satisfaction and school connectedness, regardless of ethnic group [41]. Both school connectedness and school satisfaction are factors preventing drop-out from school. [42]

A recent analysis of data from the National Longitudinal Study of Adolescent Health [20] found evidence of a positive association between PA and components of mental health, including self-esteem, emotional well-being, spirituality, and future expectations. When participation in PA/sports also included parental involvement, the behavioural risk profile became even more positive.

A cross-sectional questionnaire study of 245 Finnish adolescents [43] observed no association between PA level and school satisfaction and the trend to a weak correlation between PA level and problems at school was not statistically significant. However, PA was correlated with global school satisfaction (r = -0.21 for boys) and absence of a depressive mood state (-0.20 and -0.26 for girls and boys, respectively).

### What are the acute effects of PA on cognitive function?

Many authors have documented the acute effects of PA on cognitive function. Three recent reviews and/or meta-analyses examined these studies. [44-46] In a meta-analysis of 44 studies, Sibley and Etnier [45] concluded that PA was positively associated with better cognitive functioning in children. Some groups, particularly middle school students (grades 6–8, aged 11–13 years) and younger, seemed to benefit more from PA. Sibley and Etnier [45] noted that unpublished studies had a higher effect size than published reports, suggesting that no bias had occurred from a failure to publish non-significant results.

Brisswalter et al. [44] reviewed published studies into the effects of exercise on various tasks. They concluded that the optimal intensity for decisional tasks covered a wide range (~40–80% VO_2_max). An exercise duration of more than 20 minutes was most efficient in increasing the performance of perceptual and decisional tasks. [44,46] Tomporowki [47] suggested an upper limit of 60 minutes might arise from the adverse effects of dehydration on cognitive functions.

The literature generally suggests a positive effect of acute physical exercise on cognition. Other activities, like involvement in music also have the potential to increase reading skills, although in this case there is no positive influence on PF. [48]

### Relationship of PF with academic achievement

What is the effect of a high level of PF on academic performance? Is good cognitive functioning associated with above average PF? If so, is this a consequence of PF per se, or of better overall physical health? When analyzed globally, the literature does not indicate any clear linkage between PF and either academic achievement or intellectual performance. As early as 1969, Railo found no relationship between PF and either of these outcomes. [49] More recently, Etnier et al. [50] concluded from a meta-regression analysis that the empirical literature did not support a link between cardiovascular PF and academic achievement. However, this meta-analysis revealed a weakness in the literature: there was little data on the relationship between PF and academic achievement in school-aged children. Indeed, only 1 of the 37 studies identified included this age group.

When the definition of PF includes aspects other than cardiovascular fitness, there seems evidence of positive correlations between various measures of psychomotor performance, cognitive abilities and academic achievement. [51,52] Psychomotor performance shares many common neurological mechanisms with cognitive functions.

A 2001 cross-sectional study on California children disclosed a positive relationship between reading and mathematics results (as measured by Stanford Achievement Test-9) and results on a field test of physical fitness (the Fitnessgram). Despite a huge sample of students from grades 5, 7 and 9 (n = 954,000), potential selection biases were not considered, making it difficult to conclude that PA was linked to increased academic performance. [53] When found, any effects of PF were small. Another weak association between PF and academic achievement was observed in South Korean children (grades 5, 8, and 11); in this study, the association was much smaller than that between academic achievement and regular meal eating. [54] Dwyer et al. [24] measured muscle fitness in 9,000 Australian students. They found significant but weak associations, ranging from r = -0.10 to -0.19 for running distances of 50 m and 1.6 km, and from r = 0.10 to 0.22 for sit-ups and standing long jump, respectively.

### School sports and academic achievement

The connection between school sports and intellectual achievement has been a long-standing issue since Davis and Cooper [55] first reported a positive association between school sports participation and academic achievement. It remains the subject of recent investigations. The competitive dimension of most sports introduces particular problems, even in the school context, as the educational dimension tends to be relegated to a secondary level. The literature comprises mainly cross-sectional data and the results are more equivocal than for PA; unfortunately, most of the earlier studies did not control for biases common to athletic and academic achievements. [56,57]

Data from the longitudinal Maryland Adolescent Development in Context Study included 67% African-Americans and 33% European-Americans; it found that participation in extracurricular PA was a significant predictor of better academic results and of higher academic expectations. [58] Furthermore, sports participation by 8th grade African-American males resulted in aspirations to continue their studies toward college, with less likelihood of acting inappropriately in school. [59] In their female counterparts, sports participation also resulted in higher aspirations and in a reduction of absenteeism.

Cooper et al. [60] found that even after eliminating confounding factors, extracurricular activities, including sports and PA were predictors of better academic achievement in 2,200 American high school students. Their conclusion is in line with the point that Marsh made in 1992, that such activities may have an effect on academic achievement by increasing motivation and investment in school. [61] Another study of 11,957 American adolescents found that even after standardization for SES, sports participation with parental presence was associated with an increased probability of good grades in English and mathematics, the Adjusted Relative Risk being 1.23 for both subjects. [20] Dexter [62] examined the relationship between sports knowledge, sport performance and academic ability, the last being measured by scores on the British General Certificate of Secondary Education (GCSE). They observed a small but significant positive correlation between sports performance and GCSE score for both mathematics and English.

Melnick et al. [63] detected no relationship between academic achievement and sports participation in 3,686 African-American and Hispanic students from the "High-school and Beyond Study". However, sports participation was associated with a lower drop-out rate. Therefore, they suggested that if sports participation contributes to academic achievement, it may do so indirectly, by encouraging retention in school. Fisher et al. [64] also observed no association between sports involvement and self-reported grades in an ethnic mix of 838 grade 9 to 12 students (predominantly 63% African-American and 27% Hispanic).

Harvard students involved in varsity teams had a slightly lower GPA than their peers, but reported a higher degree of satisfaction with their university experience. [65] This also seemed the case in other institutions examined by Light. Athletes have more friends and a stronger sense of belonging to their institution. They are, according to Light, "the happiest on campus". Generally, this same trend is seen among high-school athletes. Students engaged in extracurricular PAs do not achive different academic scores than their peers, but they feel a greater engagement with their institution. [66,67] This may reflect in part the greater attention directed towards these specific students. Indeed, participants in extracurricular activities (including sports) have more interactions with significant adults than non-participants. [66]

Sport is a very complex phenomenon. There are many cultures within school sports, and any effect on academic achievement is influenced by gender, race, type of sport, type and level of athletic involvement. White and McTeer [68] suggested that the status of a given sport may influence its effect on academic achievement. Their results showed that high-status sports had a positive influence on English grades but they saw no evidenceof an effect of such sports on mathematics grades. They suggested that academic performance was more likely to be affected by cultural factors in subjective subjects like English than in mathematics. Any influence of school sports participation may also differ between girls and boys [33], and between various ethnic and cultural groups. [69]

In conclusion, the available literature suggests that sport is more likely to benefit academic achievement if offered in school rather than in other sport contexts, given the proximity of educational resources and environment. This may be particularly important for team sports, which often seem associated with risky behaviours, particularly binge drinking of alcohol. [70] When sports-involved students identify themselves as athletes rather than «jocks», such risky behaviours seem less prevalent. [67] Greater academic coaching of school athletes could be a factor favouring their academic achievement. [67] School sports should be monitored closely, with the intent of avoiding a drift away from educational objectives. It appears that satisfaction with sports vs. satisfaction with school work is predicted by a differing psychological domain (perceived ability vs. task orientation). [71] It may be helpful to create an environment where both types of endeavour find common ground, i.e. school may be the best setting in which sports can be directed towards task orientation and skills acquisition, without decreasing the pleasure and satisfaction of being good at sports and PA. As noted in various long-term follow-ups, elite and varsity level athletes later tend to experience greater educational and labour market success than non athletes. [34,67,72,73] Current evidence suggests that this effect may be mediated by racial group. [74]

### Populations with special educational needs

Academic integration of children with various behavioural and developmental problems is a growing trend in industrialized countries. The question arises in terms of their academic achievement. Reviews of exercise programmes for children with learning disabilities [75,76] have suggested that in order to increase the likelihood of positive outcomes, such programmes should have a low student-instructor ratio. Benefits (with the exception of increased PF) may reflect increased attention toward the participants.

In hyperactive impulsive children, PA is associated with global satisfaction in boys and an absence of depressive emotions in both sexes. [77] An outdoor education programme also decreased behavioural problems in children with attention deficit hyperactivity disorder. [78]

In children with reading disabilities, a school-based programme of balance and coordination training, throwing, catching, and stretching produced significant improvements in both reading and semantics. [79] Positive changes were maintained for at least 18 months following the programme, reducing the likelihood of a Hawthorne effect. [80]

Four pupils with emotional and behavioural disorders were directly studied before and after a 10-week PE intervention. Back in class, there was an increase (13.8%, or a little more than 23 minutes) in the amount of time spent focused on the tasks they were supposed to be performing. [81] A 10-week PA intervention in children with learning disabilities improved classroom behaviour and the perception of academic competence was increased. [76] However, a similar outcome was seen in the control group, indicating that there had been no specific effect from the programme.

The effects of school PA upon children with learning problems thus remains an open field for research.

### Is the potential beneficial effect of PE, school PA and sport supported by fundamental research?

The positive association observed between PA and intellectual performance among children in quasi-experimental studies should be supported by mechanistic, experimental evidence. No one can deny the important role of neurosciences in the comprehension of academic achievement. [82] Most research on the relationships between PA and cognition has centered on the hippocampus, a brain region that mediates memory and learning in mammals, and on changes in the cerebral circulation. The hippocampus has an important role in the consolidation of memory. One major mechanism essential to its functions is long-term potentiation, or LTP. LTP leads to an enhancement of nervous influx following a first series of stimuli.

#### Exercise and learning mechanisms

Hippocampal LTP is the most credible physiological explanation for learning and memory in mammals, including humans. [83] LTP leads to an increase of synaptic efficacy following an increase of synaptic traffic. [83] It was shown recently that PA favours hippocampal LTP. [84] Chronic exercise favourably influences the hippocampus through 3 mechanisms:

1) Heightened neurogenesis, i.e. an increased formation of new neurons after chronic PA, as demonstrated in the adult mouse [85,86],

2) Augmented LTP itself, i.e. enhanced neuronal transmission in the hippocampus. Different methods employed to measure cognitive functions, and scores on these tasks are well correlated with a better performing hippocampus [87]. Radial maze learning, i.e. an hippocampal spatial learning, is increased in both male and female rats exercised by voluntary running. The performance of this task does not seem to be influenced by changes in fitness of the animal, as is the case for the Morris water maze. However, if the water maze is used, it remains possible to control for an animal's level of fitness. Other studies using the Morris water maze have also reported improved performance. [85,88] Exercise has no effect on glutamate receptors in the hippocampus in aged rats [89], reinforcing the view that post-receptor mechanisms are responsible for stronger LTP in active animals. However, this point remains to be confirmed in the hippocampus of younger animals,

3) Chronic exercise creates a favourable environment for LTP by increasing the hippocampal concentrations of neuroprotective factors like brain-derived neurotrophic factor (BDNF) [90] and of other growth factors such as insulin-like growth factor (IGF-1), nerve growth factor, and fibroblast growth factor 2 (FGF-2).

The brain concentration of some antioxidants is also increased in trained animals, thus protecting hippocampal cells from oxidative damage. [91] Radak et al. [92] studied the acute effects of exercise (2 hours). Oxidative damage to macromolecules was reduced through an increase of glutathion synthetase activity and a reduction in the deleterious, inactivity-related efflux of glutamate (the neurotransmitter of learning in the hippocampus). Acute exercise also normalized certain memory functions, particularly orientation time to novelty and passive avoidance reactions.

To our knowledge, these mechanisms of enhanced learning and memory have never been explored in animals at a developmental stage corresponding to school-age children. We hypothesize that, given the higher brain plasticity of childhood, the changes seen in older brains may have an even greater magnitude in the developing brain. The data suggest that the brain structures involved in learning and memory, although more complex, function much like skeletal muscle. To enhance function (i.e. increase memory and learning), periods of stimulation must be followed by a recovery period when supercompensation can take place, and the new proteins associated with learning and memory consolidation can be synthesized.

## Discussion and Conclusion

Available data suggest that school PA (PE instruction, free time PA or school sport) could become a consistent component of PA to meet current guidelines for children and adolescents without impairing academic achievement, even if curricular time for so-called academic subjects is curtailed. In his classical work "The Adolescent Society," James S. Coleman advanced the concept of a zero-sum model. [93] This hypothesized that if time was taken from academic programmes to allow other pursuits, academic achievement would suffer. This concept may be applicable if time is spent in paid employment while attending school [94], but it does not seem to apply to extracurricular activities like sports or curricular PE. [95] In contrast, such activities are likely to increase attachment to school and self-esteem which are indirect but important factors in academic achievement.

Parents concerned about decreases in study and homework time may be better advised to question the time their children spend on TV and computer games rather than the time that they devote to PE, PA or sports in school. Indeed, the more children watch TV, the greater the decline in their academic results. [96] At least one Canadian study found that the time devoted to PA was positively associated with the time that school-aged children spent in reading. [97] Parents interested in the health and academic success of their offspring should focus on the increased prevalence of various metabolic pathologies in which sedentary behaviour plays a key etiologic role, for example, obesity and type 2 diabetes, both of which are beginning at an ever younger age. [98] Such pathologies have the potential to affect school performance adversely, although this is an area where more research is needed. [99] In one recent article, obese 3^rd ^grade girls (8 years old) did not have poorer academic results after control for SES, but relative to normal weight girls they exhibited more displaced behaviours like arguing and fighting, as well as more depressive symptoms like loneliness and sadness [100].

Engagement in PE instruction would probably be increased if grades were allocated for performance in PE, particularly in high school. The engagement of girls, particularly, decreases when PE is not considered incalculating their GPA. [101,102] However, between grade 8 and 12, the school drop-out rate for adolescents of both sexes is reduced by sport participation [103]

Another problem that remains to be resolved, despite a call for action from the Surgeon General in 1996, is the heterogeneity in provision of PE [104], extracurricular sports and other school PA programmes [105], schools with a low SES being particularly disadvantaged. School sport would appeal to more students if emphasis was placed on its educational potential rather than its competitive side. Potential drifting of objectives should be monitored to avoid a «subversion» of the educational mission and ensure a maximisation of positive effects such as academic achievement and long term adherence to physical activity. The current emphasis on a limited range of team sports should be modified to provide opportunities for students who are interested in and have the skills relevant to other sport ventures, thus attracting a wider range of students.

Many questions remain to be clarified on the relationship between academic performance, PE, school PA and sports. However, to paraphrase Eccles et al. [67], "We now know enough about the kinds of programs likely to have positive effects on children and adolescents' development." The literature strongly suggests that the academic achievement, physical fitness and health of our children will not be improved by limiting the time allocated to PE instruction, school PA and sports programmes.

## Competing interests

The author(s) declare that they have no competing interests.

## Authors' contributions

Both authors have been involved in the writing of this manuscript and have read and approved the final text.
